# Prolactin receptor is a negative prognostic factor in patients with squamous cell carcinoma of the head and neck

**DOI:** 10.1038/bjc.2011.131

**Published:** 2011-04-19

**Authors:** T Bauernhofer, M Pichler, E Wieckowski, J Stanson, A Aigelsreiter, A Griesbacher, A Groselj-Strele, A Linecker, H Samonigg, C Langner, T L Whiteside

**Affiliations:** 1 Division of Oncology, Department of Internal Medicine, Medical University of Graz, Auenbruggerplatz 15, Graz A-8036, Austria; 2 Departments of Pathology, Immunology and Otolaryngology, Hillman Cancer Center, University of Pittsburgh Cancer Institute, Pittsburgh, PA 15213-1863, USA; 3 Institute of Pathology, Medical University of Graz, Graz A-8036, Austria; 4 Research Facility for Biostatistics, Medical University of Graz, Graz A-8010, Austria; 5 Department of Oral and Maxillofacial Surgery, Medical University of Graz, Graz A-8036, Austria

**Keywords:** prolactin receptor, head and neck cancer, prognosis, growth factor, proliferation

## Abstract

**Background:**

The influence of human prolactin (hPRL) on the development of breast and other types of cancer is well established. Little information, however, exists on the effects of hPRL on squamous cell carcinomas of the head and neck (SCCHNs).

**Methods:**

In this study, we evaluated prolactin receptor (PRLR) expression in SCCHN cell lines and assessed by immunohistochemistry the expression in 89 patients with SCCHNs. The PRLR expression was correlated with clinicopathological characteristics as well as clinical outcome. The effect of hPRL treatment on tumour cell growth was evaluated *in vitro*.

**Results:**

Immunoreactivity for PRLR was observed in 85 out of 89 (95%) tumours. Multivariate COX regression analysis confirmed high levels of PRLR expression (>25% of tumour cells) to be an independent prognostic factor with respect to overall survival (HR=3.70, 95% CI: 1.14–12.01; *P*=0.029) and disease-free survival (*P*=0.017). Growth of PRLR-positive cancer cells increased in response to hPRL treatment.

**Conclusion:**

Our data indicate that hPRL is an important growth factor for SCCHN. Because of PRLR expression in a vast majority of tumour specimens and its negative impact on overall survival, the receptor represents a novel prognosticator and a promising drug target for patients with SCCHNs.

The neuroendocrine hormone prolactin (PRL) has been shown to promote growth and differentiation of several organs in vertebrates, including breast, ovaries and prostate (Hennighausen *et al*, 1997; Ahonen *et al*, 1999; Russell and Richards, 1999). In addition, human PRL (hPRL) plays an important role in physiological and pathological processes of the squamous epithelium of the human skin and can influence hair follicle growth and keratinisation (Foitzik *et al*, 2009; Ramot *et al*, 2010). Several studies have provided epidemiological data indicating involvement of hPRL in cancer development, specifically in breast, prostate and, as recently shown by our group, colorectal cancer (Tworoger *et al*, 2007; Harvey *et al*, 2008; Tworoger and Hankinson, 2008; Harbaum *et al*, 2010).

A series of experimental studies corroborate the role of hPRL in cancer biology. Upon binding to the prolactin receptor (PRLR), hPRL activates PRLR-associated Janus kinase 2 (Jak2), which in turn activates signal transducers and activators of transcription (Stats) (Hennighausen *et al*, 1997). The PRLR-activated Stats promote the growth of human prostate cancer cells by regulation of Bcl-XL and cyclin D and the survival of breast cancer cells via induction of HSP90A (Dagvadorj *et al*, 2008; Perotti *et al*, 2008). In addition to this classical PRLR-dependent signal transduction pathway, PRLR exerts tumour-promoting effects by activating numerous other molecular mechanisms. These include autocrine stimulation via the Jak-2/Stat5a/b pathway in prostate cancer (Dagvadorj *et al*, 2007), crosstalk with the PI3K/mTor pathway in lymphoma cells (Bishop *et al*, 2006), induction of vascular endothelial growth factor (Goldhar *et al*, 2005) and synergism with the insulin-like growth factor I receptor in breast cancer (Carver *et al*, 2010). Thus, given the widespread expression of PRLR in cancer, its interaction with several known signal transduction pathways and the availability of PRLR antagonists (Scotti *et al*, 2008; Tomblyn *et al*, 2009), PRLR represents an attractive drug target. Whereas the involvement of hPRL and its receptor in skin physiology and various cancer types is well documented, little is known about the role of this hormone in the pathogenesis of squamous cell carcinomas of the head and neck (SCCHNs). An initial observation connecting circulating hPRL with the development of SCCHNs came from an early report by Bhatavdekar *et al* (1993). In this study, hyperprolactinemia proved to be an independent predictor of short-term prognosis in tongue cancer patients. In a larger trial conducted by the same group, hPRL serum levels in patients with advanced tongue cancer were significantly higher compared with control subjects, and hPRL serum levels were identified as an independent prognostic factor for overall patient survival (Bhatavdekar *et al*, 2000). In contrast to these data, Meyer *et al* (2010) recently reported that circulating serum hPRL levels did not significantly predict outcome in a study of 527 patients with SCCHNs. Data regarding the role of circulating hPRL in the growth and progression of SCCHNs are contradictory, and data concerning the role of PRLR in SCCHN tissues are currently not available. Therefore, we evaluated the role of hPRL and its receptor in the pathogenesis of SCCHNs. The hypothesis tested was that hPRLR could be a potential drug target. We used immunohistochemistry (IHC) to determine the prevalence of PRLR expression in SCCHN tissue samples and assessed PRLR expression in SCCHN cell lines by immunocytochemistry, flow cytometry and immunoprecipitation. In addition, we correlated the PRLR expression in tissue samples with clinicopathological parameters and disease-free and overall survival data. Finally, we determined the effects of hPRL treatment on growth of SCCHN cells *in vitro*.

## Materials and methods

### Cell lines

The human breast cancer cell line T47D was obtained from the American Type Culture Collection (Manassas, VA, USA) and was maintained in RPMI-1640 medium (Life Technologies, Inc., Grand Island, NY, USA) supplemented with 10% (v/v) fetal bovine serum (FBS), 1 mM glutamine, 100 U ml^–1^ penicillin and 100 *μ*g ml^–1^ streptomycin (Life Technologies, Inc.). All other tumour cell lines studied were established from patients with SCCHNs at the University of Pittsburgh and were maintained in our laboratory, as described previously (Heo *et al*, 1989). Detailed characteristics of the primary tumours (designated as A) and the corresponding metastatic lesions (designated as B) are listed in Table 1. Before the proliferation assays, the PCI-6A and PCI-6B cell lines and the T47D cell line were cultured in DMEM and RPMI-1640 supplemented with 10% charcoal-stripped (CSS) FBS for 1 week, respectively. Charcoal stripping of heat-inactivated FBS was performed using 1.5 g dextran-coated charcoal (Sigma, St Louis, MO, USA) for every 100 ml FBS, with stirring overnight at 4 °C. The charcoal-stripped FBS was then centrifuged at 14 000 r.p.m. for 20 min at 4 °C, and the supernatant was recovered and sterilised utilising a 0.2-*μ*m pore size filter (Nalgene, Rochester, NY, USA). FBS was stored at −20 °C until use. A similar procedure removes at least 85% of endogenous lactogens in FBS (Biswas and Vonderhaar, 1987).

### Flow cytometry and immunocytochemistry

For measurement of PRLR expression on SCCHN cell lines, we used an unlabelled protein-A-purified mouse anti-human PRLR monoclonal antibody (mAb) B6.2 (Ab-1, Clone B6.2; Thermo Fisher Scientific, Fremont, CA, USA). The specificity of this reagent has been reported previously in the T47D cell line (Banerjee *et al*, 1993). Details of flow cytometry and immunocytochemistry methods are described in the Supplementary Information online. The cells were analysed using FACScan (Becton Dickinson, Mountain View, CA, USA) and Lysis II software. For each sample, 10 000 events were acquired, and the cell number and mean fluorescence intensity were determined.

### Immunoprecipitation

To investigate the specificity of PRLR antibody binding, we performed PRLR immunoprecipitation using PCI-6A, PCI-6B and T47D cells. For details of this analysis, please see also in the Supplementary Information online.

### Patients

The PRLR expression was assessed in tissues of two independent patient cohorts. The preliminary study was performed using a group of 13 SCCHN samples that were randomly selected from tumour tissue archived in the Department of Pathology, University of Pittsburgh, USA. In addition, tissue sections of the primary tumour and lymph node metastases, from which the PCI-6A and PCI-6B cell lines had been generated, were studied. Tissue staining was performed as described previously (Mertani *et al*, 1998). All tissues and human cell lines used in the experiments were obtained from consenting subjects under the IRB approval from the University of Pittsburgh Cancer Institute.

To evaluate the prognostic value of PRLR expression in SCCHN and to further substantiate the results of our preliminary study, a larger cohort containing 89 SCCHN specimens was studied using tumour tissue archived at the biobank of the Medical University Graz, Austria. This retrospective study included randomly selected patients diagnosed with SCCHN in the oral cavity between January 1992 and December 2002. For each specimen, both primary tumour tissue and clinicopathological data were available. Patients did not receive neoadjuvant therapy, and all underwent curative resection. The median follow-up period was 5.8 years. For each patient, postoperative surveillance was performed including routine clinical and laboratory examination every 3 months, and CT scans of the head and neck as well as X-rays of the chest every 6 months. After 5 years, intervals were extended to 12 months. The 6th edition of the American Joint Committee on Cancer (AJCC)/International Union Against Cancer (UICC) TNM system was used to classify the patients. The characteristics of the patients are shown in Table 2.

### Immunohistochemistry

We performed immunohistochemistry using a tissue microarray (TMA) technique, as described previously (Kononen *et al*, 1998). Briefly, TMAs were constructed using a manual tissue-arraying instrument (Beecher, Silver Spring, MD, USA). To account for tumour heterogeneity, three cylindrical core biopsies, 0.6 mm in diameter, were excised from various sites within each tumour and arrayed on a recipient paraffin TMA block. The TMA sections (4 *μ*m) were stained using an automated staining system (BenchMarkTM; Ventana Medical Systems, SA, Illkirch, CEDEX, France). Tissue digestion was performed using Protease Type I (0.5 units ml^–1^; catalogue no. 760-2018, Ventana) for 32 min. The primary PRLR antibody (Ab-1, Clone B6.2; Thermo Fisher Scientific) was applied in a 1 : 400 dilution, and the reaction was visualised using the ultraVIEW Universal DAB Detection KitTM (catalogue no. 760-500; Ventana). Immunoreactivity was independently assessed by two experienced pathologists (CL and AA), who were blinded to clinicopathological data, using a semiquantitative scoring system. Discrepancies were resolved by simultaneous re-examination on the slides by both investigators using a double-headed microscope. Membranous and/or granular cytoplasmic staining was considered positive, and immunoreactivity was semiquantitatively categorised as follows: focal (<25% of tumour cells), moderate (25–75% of tumour cells) or extensive (>75%). For analysing the prognostic value of PRLR expression in SCCHNs, we defined the focal expression pattern as the low PRLR expression group and summarised tumours with moderate and extensive PRLR expression to a high PRLR expression group. Each tumour was scored assessing the average positivity of the core biopsies. Samples of breast cancer tissue known to express PRLR served as positive control. Negative controls included omission of the primary antibody and incubation with the Ventana Antibody Diluent (catalogue no. 251-018).

### MTT assay

Tumour cell lines were cultured in the presence or absence of various concentrations of hPRL at 20, 40 or 100 ng ml^–1^; kindly provided by Dr AF Parlow, National Hormone & Peptide Program (Torrance, CA, USA). Cell proliferation was measured using the colorimetric MTT (3-(4,5-dimethylthiazal-2-yl)-2,5-diphenyltetrazolium bromide) assay, as previously described (Lin *et al*, 1995).

### Statistical analysis

All statistical analyses were performed using SPSS version 17.0 software (SPSS Inc., Chicago, IL, USA). The Fisher's exact test, *χ*^2^ test and the Mann–Whitney procedure were used to analyse PRLR expression in relation to each clinicopathological parameter. The disease-free and overall survival of the patients were calculated using the Kaplan–Meier method and compared by the log-rank test. Backward stepwise Cox proportion analysis was performed to determine the influence of PRLR expression, T and N classification, tumour grade, patient age and gender on overall and disease-free survival. Hazard ratios (HRs) estimated from Cox models were reported as relative risks with corresponding 95% confidence intervals (CIs). The assumption of proportional hazards was checked by LML plots and residual analysis using Schoenfeld plots. A *P*-value of <0.05 was considered significant.

## Results

### PRLR expression in SCCHN cell lines

**Figure 1:**
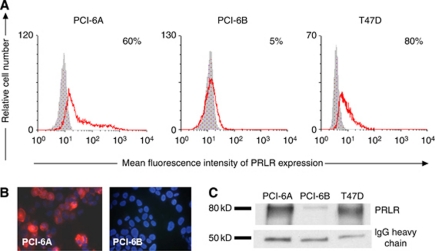
PRLR expression in SCCHN cell lines. (**A**) PRLR expression measured by flow cytometry in PCI-6A, PCI-6B and T47D cell lines. The PCI-6A cell line shows a significantly higher percentage of PRLR+ cells compared with the PCI-6B cell line. (**B**) Immunocytochemical staining of SCCHN cell lines (magnification × 400). Positive membranous and cytoplasmic PRLR staining (red colour of Cy3) is shown for the PCI-6A cell line, whereas the PCI-6B cell line is negative. Nuclei are counterstained with Hoechst dye (blue). (**C**) Immunoprecipitation reveals a prominent band between 75 and 80 kD, corresponding to the PRLR protein in the lysate of PCI-6A cells, whereas only a weak band was detected in the lysate of PCI-6B cells. Immunoglobulin G (IgG) heavy chain was used as a loading control and T47D cell line served as a positive control.

**Figure 2:**
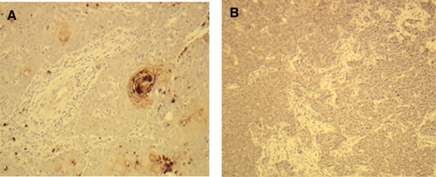
PRLR expression in SCCHN tissues. In agreement with the corresponding cell lines PCI-6A and PCI-6B, the primary tumour (**A**) shows PRLR positivity, whereas the metachronously developed neck lymph node metastasis (**B**) is PRLR negative.

The PRLR expression was initially assessed in SCCHN cell lines (*n*=8) established from primary tumours and the corresponding metastases from four patients. The breast cancer cell line T47D served as the positive control. The PRLR expression was determined in the SCCHN lines by flow cytometry and immunocytochemistry. Of the eight SCCHN lines, five (4A, 4B, 6A, 37A, 37B) were positive for PRLR expression (Table 3). The only pair of SCCHN cell lines with a heterogeneous PRLR expression profile was PCI-6A and PCI-6B (Figure 1A). The majority of PCI-6A cells, which had been generated from a primary tumour, were highly PRLR positive, whereas all PCI-6B cells generated from a metachronously developed corresponding lymph node metastasis were negative for PRLR, both on the cell surface and in the cytoplasm (Figure 1B). To substantiate the specificity of PRLR staining results, we examined PRLR expression in the SCCHN cell lines PCI-6A and PCI-6B using immunoprecipitation. In accordance with data obtained from immunocytochemistry and flow cytometry, PRLR immunoprecipitation resulted in a prominent band at 80 kDa that corresponded to the PRLR-predicted molecular weight using lysate from PCI-6A cells, whereas only a weak band was detected in PCI-6B cell lysate (Figure 1C). To exclude the possibility that the divergent PRLR expression profiles of PCI-6A and PCI-6B cells might be because of culture selection, we evaluated PRLR expression in sections of the primary tumour and the metachronously developed lymph node metastasis by immunohistochemistry (Figure 2). The results from these samples confirmed the data obtained from the cell lines. To further validate the results obtained with the SCCHN lines, we examined 13 tissue specimens of primary and metastatic SCCHNs randomly selected from the institutional tumour tissue archive. Breast tissue specimens known to express PRLR served as control. Of 13 SCCHN specimens, 9 (69%) were positive for PRLR expression based on the intensity of PRLR staining and the distribution of positive cells (data not shown).

### PRLR expression and correlation with clinicopathological parameters and patients’ outcome

**Figure 3:**
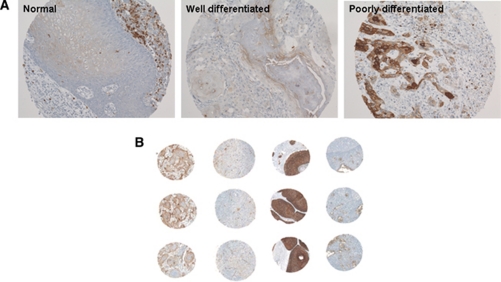
Immunohistochemistry for PRLR in SCCHN tissues. (**A**) Note low PRLR expression level in normal tissue and well-differentiated tumour tissue, and high PRLR expression level in poorly differentiated tumour tissue ( × 100). (**B**) PRLR expression on representative SCCHN spots on the TMA comprising three paired spots (columns) from four different tumours (lines). Note the variable PRLR immunoreactivity between different tumours (lines) and the high concordance between different cores from the same tumour (columns).

**Figure 4:**
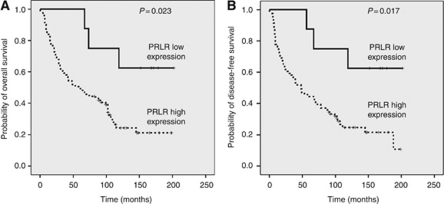
Kaplan–Meier plots for overall survival (**A**) and disease-free survival (**B**) in patients with SCCHNs with low *vs* high PRLR expression.

To evaluate whether PRLR expression is associated with the outcome of patients with SCCHN, we determined PRLR expression by immunohistochemistry in a larger set of human SCCHN tumour samples. Expression of PRLR was observed in 89 primary tumours with 8 (9%) cases showing focal, 16 (18%) moderate and 65 (73%) extensive immunoreactivity, respectively (Figure 3). Median follow-up was 70 months (mean 73, range 1–202). The 5-year disease-free survival was observed in 43 (48%) patients, 46 (52%) patients developed disease recurrence including 6 (7%) patients who developed distant metastasis and 41 (46%) died during a 5-year follow-up period. A comparison of the moderate PRLR expression group (25–75%) with the extensive PRLR expression group (>75%) showed no differences for clinical outcome in the univariate analysis. Therefore, we compared focal expression (<25%) (categorised to the PRLR low expression group) *vs* moderate and/or extensive expression (categorised to the PRLR high expression group) and found that a higher degree of PRLR expression was associated with decreased disease-free and overall survival. After a cutoff value of 25% positive tumour cells had been identified as the strongest discriminator for patient outcome, all subsequent analyses used this value, and we defined tumours showing <25% PRLR-positive tumour cells as the PRLR low expression group and tumours showing >25% PRLR-positive tumour cells as the PRLR high expression group. Over the complete follow-up period, the rate of disease recurrence (75 *vs* 38%, *P*<0.05), including distant metastases (7 *vs* 0%), was higher in the PRLR high expression group compared with the PRLR low expression group. Also, 58 out of 81 (72%) patients with tumours with high PRLR expression and 3 out of 8 (38%) patients with tumours showing low PRLR expression died during the follow-up period (*P*=0.023, log-rank test; Figure 4A). Disease-free survival after 5 years was documented in 36 out of 81 (44%) patients with tumours with high PRLR expression and 7 out of 8 (87%) patients with tumours showing low PRLR expression (*P*=0.017, log-rank test, Figure 4B). Furthermore, T and N classification, both known to define a poor prognosis in SCCHNs, were significantly associated with disease-free and overall survival (data not shown). No association between PRLR expression and clinicopathological parameters, such as T and N classification as well as tumour differentiation, gender and age, was observed (Table 4). To determine the independent prognostic value of PRLR expression on patients’ outcome, a multivariate analysis using a Cox proportional hazard model was performed. In Cox’s proportional hazards regression models including PRLR expression, T and N classification, tumour grade, as well as patient age and gender, we identified advanced tumour stages (stage I and II *vs* III and IV; HR=1.78, 95% CI: 1.03–3.05; *P*=0.034), nodal positive disease (nodal negative *vs* nodal positive disease; HR=2.20, 95% CI: 1.30–3.75; *P*=0.003) and high PRLR immunoreactivity (low *vs* high expression group; HR=3.70, 95% CI: 1.14–12.01; *P*=0.029) as independent prognostic variables with respect to overall survival. For the other parameters tested, no independent influence on outcome was observed.

### hPRL effects on PCI-6A, PCI-6B and T47D cell growth

The MTT assay was used to investigate the effects of hPRL on PRLR-positive PCI-6A and PRLR-negative PCI-6B cell growth when cultured in media supplemented with CSS FBS with and without the addition of hPRL. The T47D breast cancer cell line served as positive control. We observed a moderate, although statistically significant, hPRL-dependent increase in the growth of T47D cells and PCI-6A cells when compared with untreated cells (*P*<0.01 and *P*<0.04, respectively). A 20% increase in the growth was observed for the 20 ng ml^–1^ hPRL concentration with a decline at the highest concentrations of the hormone (100 ng ml^–1^). The hPRL treatment did not affect the growth of PRLR-negative PCI-6B cells (data not shown).

## Discussion

It has been known for decades that hPRL, a peptide hormone secreted by the anterior pituitary, is a potential growth and differentiation factor for mammary epithelium. Evidence from numerous *in vivo* and *in vitro* studies has linked this classical hormone to a series of other physiological processes. Thus, hPRL may be synthesised as a paracrine/autocrine cytokine, and expression of its receptor is not restricted to the mammary gland (Ben-Jonathan *et al*, 1996; Dorshkind and Horseman, 2000). Beyond the physiological effects of this circulating hormone, a substantial number of studies focussed on the importance of hPRL in cancer biology (Ben-Jonathan *et al*, 2002; Clevenger, 2003). This approach is promising as PRLR inhibition could serve as a novel strategy for the treatment of cancer (Clevenger *et al*, 2008). To date, several lines of evidence suggest the involvement of the hPRL/PRLR axis in breast, prostate and colorectal cancer development (McHale *et al*, 2008; Harbaum *et al*, 2010). However, the role of hPRL in SCCHNs remains largely unknown. A recent study reported that the serum prolactin level was not a prognostic factor for predicting outcome in a large cohort of SCCHN patients (Meyer *et al*, 2010). Given the negative findings between circulating hPRL and prognosis in SCCHNs, we decided to study the expression of PRLR within SCCHN cancer tissue. There are three main findings in this study concerning hPRL/PRLR and SCCHNs: (1) PRLR is widely expressed in SCCHNs; (2) high level of PRLR expression is an independent negative prognostic factor for overall survival in patients with SCCHNs; and (3) hPRL acts as a growth factor for PRLR-expressing SCCHN cancer cells. We found that >90% of primary tumours included in our study were positive for PRLR, but its expression was variable, ranging from very low to high. Patients expressing high levels of PRLR had a significantly lower overall survival. To our knowledge, only two other studies have addressed the presence of PRLR in SCCHNs. In the first, Patel *et al* (1994) reported on PRLR expression in 25 male patients with advanced tongue cancer. Similar to our study, the authors found no correlation between PRLR status and any of the tested clinicopathological variables. They reported PRLR negativity in 17 patients with hyperprolactinaemia as an independent predictor for short-term prognosis. However, they used a different cutoff level for PRLR positivity and included only 25 patients in their study, which may have been insufficient to detect clinically significant differences. In the second study, PRLR measured by a ligand binding assay and by PRLR mRNA using RT–PCR were detectable in 8 out of 24 (33%) and 41 out of 50 (82%) of tumour samples, respectively. However, follow-up data, including clinical outcome, were not available in that investigation (Bhatavdekar *et al*, 2000). Our study shows for the first time that in a large cohort of patients, PRLR expression in tumour tissues is significantly associated with a decreased survival in SCCHN patients. We identified 25% positive tumour cells as a cutoff value for optimal discrimination between good and poor prognosis. However, this threshold should undergo external validation in large prospective studies, as one limitation of our study is the relative small number of patients in the PRLR low expression group. The molecular mechanisms underlying this result have yet to be clarified. Bhatavdekar *et al* (2000) detected hPRL protein and mRNA expression in 44 and 85% of SCCHN patients, respectively, and proposed a role for hPRL as a local growth promoter. However, there is currently no study available that investigates the influence of hPRL on the growth of SCCHN cancer cells generated from primary tumours and their corresponding metastases. Therefore, we selected a PRLR-positive and a negative cell line to determine the effects of hPRL on cell growth. Both the T47D breast cancer line and the PRLR-positive PCI-6A SCCHN cell line showed a moderate but significant increase of cell growth in the presence of hPRL. Similar to many cytokines that induce receptor dimerisation, hPRL exhibited a bell-shaped curve rather than a linear dose-response relationship; this is in agreement with previous reports using other cell lines (Fuh and Wells, 1995; Hooghe *et al*, 1998). This observation supports the hypothesis that hPRL might act as a local paracrine/autocrine growth-promoting factor in SCCHN. We found a significantly higher rate of local disease recurrence and a higher rate of distant metastases in the group with PRLR high expression. Therefore, it is reasonable to hypothesise that tumours expressing higher levels of PRLR might recur more frequently under the influence of local PRL exposure or exhibit a more aggressive behaviour including development of distant metastasis more frequently. In contrast to a lack of data for SCCHNs, several studies have demonstrated that hPRL acts as a growth factor for mammary epithelial cells (Clevenger *et al*, 2009). For example, hPRL promotes survival of mammary epithelial cells via activation of Akt 1 (Creamer *et al*, 2010). In addition, it decreases the phosphorylation of serine residues on the PRLR, thus impairing PRLR turnover and ultimately leading to increased hPRL signalling (Plotnikov *et al*, 2009). Finally, hPRL stimulates cell proliferation and migration by upregulation of sphingosine kinase-1 expression (Doll *et al*, 2007). However, it remains largely unknown whether these and other previously proposed molecular mechanisms are relevant to SCCHNs. Nevertheless, our experiments suggest that PRLR has a potential as a drug target in SCCHNs. Further experiments are necessary to confirm this hypothesis. Traditional agents that block pituitary hormone production do not affect extrapituitary hPRL expression. Therefore, PRLR antagonists remain the most promising novel drugs with the potential to disrupt hPRL/PRLR signalling. Howell *et al* (2008) demonstrated that the PRLR antagonist *δ*1-9-G129R-hPRL potentiates the cytotoxicity of paclitaxel in breast cancer cells. In a different study, it was shown that hPRL confers resistance to cisplatin by upregulation of the detoxification enzyme glutathione-*S*-transferase (LaPensee *et al*, 2009; Tomblyn *et al*, 2009). reported that three PRLR antagonist-fusion proteins effectively prevented tumour recurrence and development of distant metastases in a breast cancer xenograft model, a strategy that has been successfully employed for PRLR-expressing breast cancer cells (Langenheim and Chen, 2005; Tomblyn *et al*, 2009). Hence, these inhibitors may also provide a potential treatment strategy for patients with PRLR-positive SCCHN. Although a detailed review of these studies in breast cancer is beyond the scope of this article, we refer interested readers to a recent excellent review by Carver *et al* (2009) for more information. In conclusion, the present study indicates that PRLR is widely expressed in SCCHNs and that high expression of PRLR in tumour tissue is an independent negative prognostic factor for overall survival in SCCHN patients. Furthermore, our data suggest that human hPRL is a growth factor for SCCHNs and that it promotes the proliferation of PRLR-positive cancer cells. Further preclinical studies are warranted for the validation of PRLR as a novel drug target in SCCHNs.

## Figures and Tables

**Table 1:** Pathological characteristics of the primary and metastatic squamous cell carcinomas of the head and neck from which the respective cell lines were generated

**Cell line**	**Tumour/metastatic site**	**Differentiation**
PCI-4A	Larynx	Moderate
PCI-4B	Neck node	Moderate
PCI-6A	Tonsil	Well–moderate
PCI-6B	Neck node	Poor
PCI-15A	Piriform sinus	Poor
PCI-15B	Lymph node	Poor
PCI-37A	Larynx	Well–moderate
PCI-37B	Lymph node	Well–moderate

**Table 2:** Clinicopathological characteristics of the SCCHN patients included in this study

**Clinicopathological parameters**	**Patients (*n*=89)**	**Proportion**
*Gender*		
Male	67	75%
Female	22	25%
		
*Median age at diagnosis*		
Male	56 (37–88)	
Female	65 (40–88)	
		
*Tumour size*		
T1	37	41%
T2	21	24%
T3	10	11%
T4	21	24%
		
*Nodal status*		
N0	60	67%
N1	14	16%
N2	15	17%
		
*Grade*		
G1	18	20%
G2	46	52%
G3	25	28%
		
*Stage*		
I	34	38%
II	16	18%
III	12	14%
IV	27	30%

Abbreviation: SCCHN=squamous cell carcinoma of the head and neck.

**Table 3:** Surface and intracytoplasmatic expression of the PRLR on four pairs of squamous cell carcinoma lines generated from the primary tumour and autologous lymph node metastases^a^

	**PRLR surface expression**		**PRLR intracellular expression**		**ICC**
**Cell line**	**% Pos. cells**	**MFI**	**% Pos. cells**	**MFI**	
PCI-4A	1	17	7	19	Pos
PCI-4B	6	38	16	21	Pos
PCI-6A	58	633	51	475	Pos
PCI-6B	1	48	1	38	Neg
PCI-15A	0	0	5	67	Neg
PCI-15B	0	0	2	80	Neg
PCI-37A	26^b^	26^b^	96	27	Pos
PCI-37B	82	640	95	435	Pos

Abbreviations: PRLR=prolactin receptor; MFI=mean fluorescence intensity; ICC=immunocytochemistry; Pos.=positive.

a Data were obtained by flow cytometry of dissociated monolayers or by ICC of cytocentrifuged cells.

b After trypsinization that led to considerable loss of PRLR surface expression but cells could not be detached from culture flask otherwise.

**Table 4:** Correlations between PRLR expression and clinicopathological parameters

	**PRLR immunoreactivity**		
**Clinicopathological variables**	**PRLR low expression**	**PRLR high expression**	***P*-value**
*T classification*			
T1	2 (25%)	35 (43%)	0.511
T2	3 (37.5%)	18 (22%)	
T3	1 (12.5%)	9 (11)	
T4	2 (25%)	19 (24%)	
			
*N classification*			
N0	7 (87.5%)	53 (65%)	0.195
N1–3	1 (12.5%)	28 (35%)	
			
*Tumour grade*			
G1	3 (37.5%)	15 (19%)	0.404
G2	3 (37.5%)	43 (53%)	
G3	2 (25%)	23 (28%)	
			
*Stage*			
I	2 (37.5%)	32 (40%)	0.868
II	3 (37.5%)	13 (16%)	
III	1 (12%)	11 (14%)	
IV	2 (25%)	25 (31%)	
			
*Gender*			
M	5 (62%)	62 (76%)	0.310
F	3 (38%)	19 (24%)	
			
*Age*			
<60	4 (50%)	44 (54%)	0.551
>60	4 (50%)	37 (45%)	

Abbreviation: PRLR=prolactin receptor.
