# Supplementary material for: Prolactin receptor is a negative prognostic factor in patients with squamous cell carcinoma of the head and neck
Source: Br J Cancer. 2011 Apr 19;104(10):1641–8. doi: 10.1038/bjc.2011.131 (PMC3101909; doi:10.1038/bjc.2011.131)
Supplement: Supplementary Information [file bjc2011131x1.doc]

**Supporting Information**

Flow cytometry

We used an unlabeled protein-A-purified mouse anti-human prolactin receptor monoclonal antibody (mAb) B6.2 (Ab-1, Clone B6.2; Thermo Fisher Scientific, Fremont, CA, USA).

Expression of PRLR was determined in permeabilized and non-permeabilized cells. Before PRLR staining, tumour cells were dissociated from plastic using a cell dissociation solution (Sigma). The cells were washed twice in phosphate-buffered saline (D-PBS, GIBCO) containing 0.1% (w/v) sodium azide, and 0.1% (w/v) bovine serum albumin (wash buffer). Cell suspensions were then adjusted to a concentration of 0.5x10^6^ cells/100 µl. For surface staining, cells were incubated with B6.2 or a mouse IgG1 isotype control (DAKO Corp., Carpintera, CA, USA) for 30 min at 4ºC. All cells were washed twice in wash buffer and incubated with pre-titered FITC-conjugated goat anti-mouse IgG F(ab)2 fragments (Caltag, San Francisco, CA, USA) for 30 min at 4ºC. After two washes, all cells were fixed with 0.5% (w/v) paraformaldehyde in D-PBS. Optimal working dilutions of the primary antibody (10 μg/ml) and secondary antibody (50 μg/ml) were determined in preliminary titration experiments. As control, samples were not stained with either the primary or secondary antibodies. The cells were analyzed using FACScan (Becton Dickinson, Mountain View, CA, USA) and Lysis II software. Ten thousand events were acquired for each sample, and the cell number and mean fluorescence intensity were determined.

### Immunocytochemistry

T47D and SCCHN cell lines were cultured on round cover slips in 12-well plates (Costar, Corning, NY, USA). When the cultured cells were 70-80% confluent, the culture medium was removed and the cells were washed three times with PBS for 5 min. In addition, cytospins of the respective cell lines were prepared after detaching the cells from the plastic with the cell dissociation solution. The cells were fixed with acetone/methanol (1:1) for 2 min at RT and then stained with mouse anti-human PRLR mAb B6.2 diluted 1:250 (1 mg/ml stock solution) with D-PBS containing 1% BSA for 1 h at RT. Afterward, cells were washed three times with PBS for 5 min and stained with Cy3-conjugated goat-anti-mouse IgG (Jackson Immuno Research, West Grove, PA, USA) diluted 1:800 in D-PBS containing 1% BSA for 1 h at RT. An isotype-specific mouse IgG1 antibody (Dako) and a sample without the primary antibody incubation were used as controls in each experiment. After three washes, Hoechst dye 33342 (2 μg/mL; Sigma) was used to counter-stain cell nuclei. Cover slips were mounted on microscope slides (Fisher Scientific, Pittsburgh, PA, USA) with fluorescent mounting medium (Dako) and viewed under a fluorescent microscope (UFX-2A, Nikon, Japan).

Immunoprecipitation

5 x 10^6^ cells were lysed, and the resulting lysate was pre-cleared with protein G-coated Sepharose beads (Sigma) for 2 h. Following centrifugation, the pre-cleared supernatants were incubated with the PRLR antibody (Ab-1, Clone B6.2; Thermo Fisher Scientific) or with a non-specific mouse anti-human IgG1 mAb overnight at 4°C and repeated. Lysates were then centrifuged and the pellets were washed three times with the following buffers: once with a PBS/1% NP-40 solution containing Tris-LiCl and twice with Tris-NaCl-ETDA. The samples were then boiled and protein was separated on a 12% Tris-HCl gel (Bio-Rad, Hercules, CA, USA) under reducing conditions. Protein was then transferred to PVDF membranes (Millipore, Bedford, MA, USA). Membranes were initially incubated in a 3% nonfat dried milk and 1% BSA solution overnight at 4°C. Membranes were then incubated anti-PRLR monoclonal antibody (1:250 dilution) at room temperature for 1 h. A washing step was performed in Tris-buffered saline supplemented with 0.05% Tween-20, and the membrane was incubated with horseradish peroxidase-conjugated anti-mouse immunoglobulin (1:5000 dilution; Amersham, Piscataway, NJ, USA) for 1 h. Protein was detected using enhanced chemiluminescence. The T47D cell line was used as positive control, and supernatant from the cell cultures and cell culture media served as negative controls. The molecular weight of each band was determined using a SeeBlue Plus2 pre-stained protein standard (Invitrogen).
